# K-FluDB: a novel K-mer-based database for enhanced genomic surveillance of Influenza A viruses

**DOI:** 10.1093/bioadv/vbaf254

**Published:** 2025-10-10

**Authors:** Alejandro Uscanga Junco, Lorena Díaz-González, Blanca Taboada

**Affiliations:** Doctorado en Ciencias, Instituto de Investigación en Ciencias Básicas y Aplicadas (IICBA), Universidad Autónoma del Estado de Morelos, Cuernavaca, Morelos 62210, Mexico; Centro de Investigación en Ciencias, Universidad Autónoma del Estado de Morelos, Cuernavaca, Morelos 62210, Mexico; Departamento de Genética del Desarrollo y Fisiología Molecular, Instituto de Biotecnología, Universidad Nacional Autónoma de México, Cuernavaca, Morelos 62210, Mexico

## Abstract

**Motivation:**

Influenza A viruses frequently cause seasonal outbreaks and pandemics due to their genetic diversity and reassortment potential. Existing genomic surveillance tools face challenges with redundant databases, delaying subtype identification and obscuring reassortment dynamics. K-FluDB, a novel k-mer-based database, addresses these issues by enhancing subtype identification, capturing genomic diversity, and assisting in the detection of reassortment events critical for understanding viral evolution and improving outbreak proactive measures.

**Results:**

K-FluDB provides a comprehensive pangenome for Influenza A, including complete and subtype-specific subsequences from 50 subtype combinations across all 18 hemagglutinin (HA) and 11 neuraminidase (NA) subtypes. Achieving 99.64% compression, K-FluDB eliminates redundancy while preserving essential information. Validation with real-world datasets showed high recovery indices (up to 96.24%) and correct subtype prediction ratios (exceeding 99% for HA and NA). K-FluDB also assists in the detection of reassortment events.

**Availability and implementation:**

Three versions of K-FluDB, optimized for read lengths of 75, 150, and 300 nucleotides, are freely available at https://zenodo.org/records/17203072, and the source code is available at https://github.com/usjunco/pangen.

## 1 Introduction

Influenza A viruses are well-known for their capacity to trigger recurrent seasonal outbreaks and, occasionally, pandemics, resulting in significant morbidity and mortality, particularly in children under 5 years of age. These viruses are classified into subtypes based on two surface glycoproteins: hemagglutinin (HA) and neuraminidase (NA). Currently, a total of 18 HA subtypes and 11 NA subtypes have been identified (Chakraborty and Chauhan 2024), forming the basis of the nomenclature of Influenza A viruses, such as H1N1 and H3N2. According to the NCBI taxonomy, a total of 131 subtypes has been acknowledged. Historical zoonotic transmissions of influenza viruses, characterized by their spread from animal hosts to humans, have resulted in severe pandemics, as observed with the H2N2 pandemic in 1957, the H3N2 pandemic in 1968, and the H1N1 pandemic in 2009 (Ding *et al.* 2021); the latter commonly known as “swine flu.” The global impact of the 2009 H1N1 pandemic, which emerged from a new reassorting virus containing genes from swine, avian, and human influenza strains, caused substantial illness and death.

Genomic surveillance of viruses has become a crucial tool for understanding their evolution and dispersion (Hill *et al.* 2023, Jung *et al.* 2024). Monitoring zoonotic strains of influenza is crucial for the early detection of potentially dangerous variants. Furthermore, annual monitoring of circulating influenza viruses is also critical to effectively establish strategies for the prevention and control of influenza at national and global levels (Andrew *et al.* 2023, Caini *et al.* 2024). In recent years, significant progress has been made in the molecular study of influenza viruses due to the introduction of high-throughput sequencing technologies, enabling rapid whole-genome sequencing. These studies provide unprecedented perspectives into the discovery of new strains, their evolutionary trajectories, antigenic properties, and genetic diversity (Vo *et al.* 2023, Liang *et al.* 2023, Tisza *et al.* 2024). Tools such as INSaFLU (Borges *et al.* 2018) have facilitated genomic analysis of influenza virus, enabling rapid identification of circulating strains and their antigenic characterization (Ghorbani *et al.* 2023, López-Valiñas *et al.* 2023, Han *et al.* 2024).

However, the effectiveness of genomic surveillance relies heavily on comprehensive and non-redundant reference databases. Redundancy in the influenza database might obscure the identification of novel subtypes, making it harder to distinguish them from existing strains. Moreover, analyzing large and redundant datasets can slow the process of identifying new subtypes due to the increased computational burden, thereby delaying critical findings. On the other hand, relying on a single reference genome may result in significant genomic variation being overlooked, leading to incomplete sequence diversity (Ren *et al.* 2020, Smith *et al.* 2022, Chorlton 2024).

This article presents K-FluDB, a novel database designed to address these challenges by offering distinct subsequences specific to all 18 HA and 11 NA segments. This database offers significant improvements in both time efficiency and specificity for genomic surveillance of influenza viruses. By constructing a subset of subtype-specific reference sequences using short genome subsequences (k-mers), K-FluDB allows for more precise subtype identification in high-throughput sequencing data. Furthermore, it provides a comprehensive exploration of genomic variation while minimizing biases introduced by redundant reference genomes. This database also facilitates the detection of novel HA and NA recombinants by enabling the identification of reads that align with subtype-specific regions within individual HA or NA segments. In addition, K-FluDB can facilitate the recognition of novel HA/NA subtype constellations not previously reported, which may represent new reassortant strains involving the surface glycoprotein segments. While most surveillance efforts focus on HA and NA segments, the remaining six genomic segments encode proteins critical for replication, host adaptation, immune evasion, and antiviral resistance. Ignoring these internal segments can obscure reassortment dynamics and underestimate evolutionary complexity. By enabling the identification of subtype-specific sequences across all segments, K-FluDB offers a comprehensive and scalable solution for influenza genomic surveillance.

## 2 Methods

### 2.1 Benchmark influenza database

To establish a comprehensive database for differentiating *Alphainfluenzavirus influenzae* (Influenza A) and its subtypes, the complete dataset of influenza virus sequences and metadata were downloaded from the NCBI Virus FTP site (accessed 8 May 2022). From this dataset, only sequences corresponding to Influenza A virus (taxon ID: 11320) were retained. Furthermore, only sequences annotated at the subtype level, defined as taxonomic nodes below the species level (e.g. H1N1, taxon ID: 114734 or H3N2, taxon ID: 115569) were included for further analysis. This dataset comprised a total of 895 900 Influenza A sequences. To reduce redundancy and preserve subtype, temporal, and segmental diversity, sequences were grouped by collection year, genomic segment, and subtype annotation. A maximum of 30 sequences was randomly selected from each group, resulting in 8998 groups and a final dataset of 81 262 sequences.

To enhance subtype differentiation, sequence compression was applied separately to each segment. Table 1 provides an overview of the sequence and nucleotide counts per segment.

**Table 1.** Initial number of sequences and nucleotides per segment after subsampling of the Influenza A dataset.

Segment	Initial	
	Seqs^a^	nt^b^
1 (PB2)	9811	21 962 041
2 (PB1)	9946	22 239 834
3 (PA)	9868	21 121 062
4 (HA)	11 287	18 222 760
5 (NP)	9872	14 841 330
6 (NA)	10 478	14 547 376
7 (M1, M2)	10 056	9 902 780
8 (NS1, NEP)	9944	8 479 502

a Seqs, number of sequences.

b nt, nucleotide count.

### 2.2 Pangenome generation

**Figure 1.**
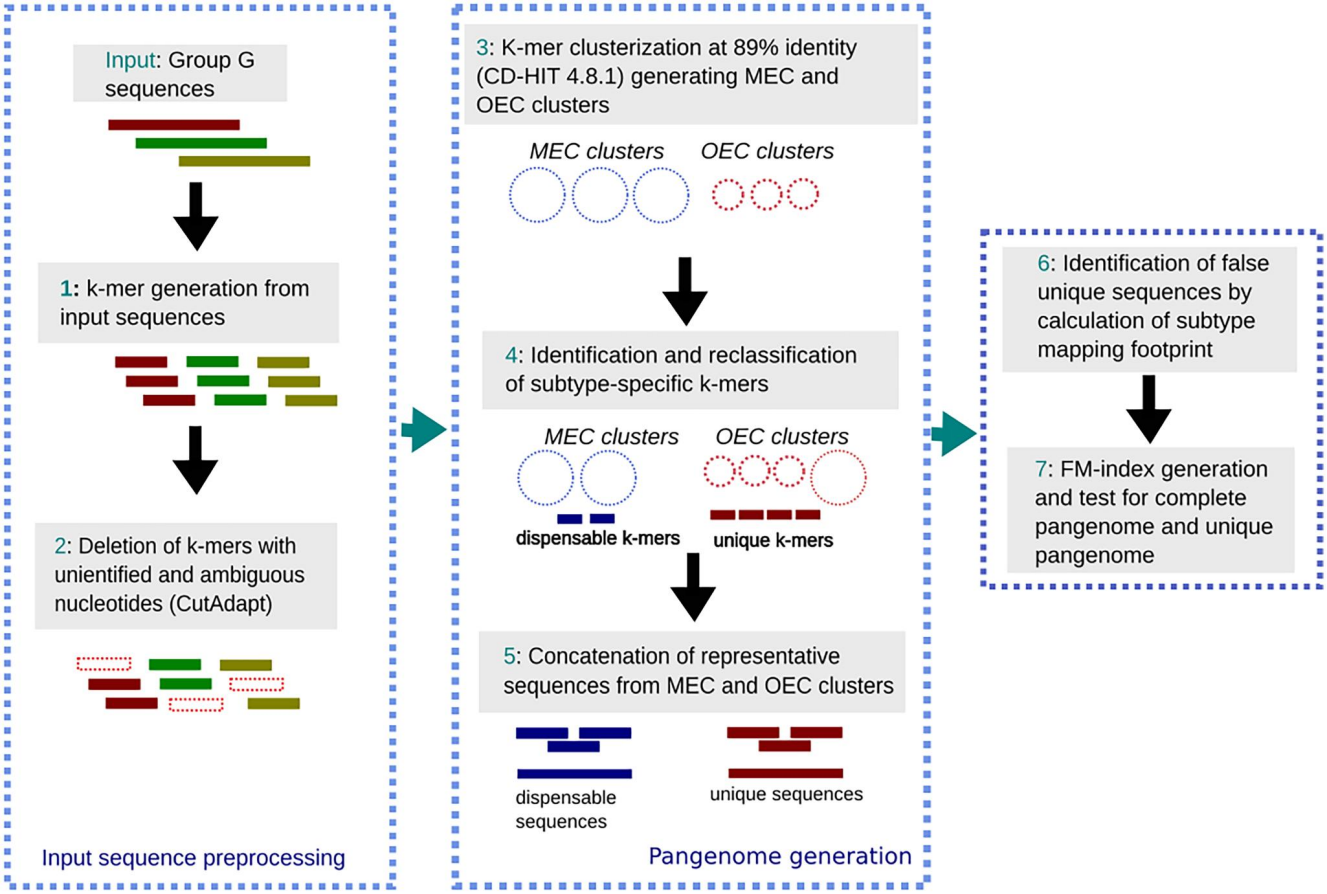
Overview of the pangenome generation workflow.

Figure 1 illustrates the pangenome generation workflow. The process begins with input sequence preprocessing, where k-mers are generated and those with unidentified or ambiguous nucleotides are removed (steps 1 and 2). Next, k-mers are clustered into multiple-element clusters (MEC) and one-element clusters (OEC) (step 3). False MEC clusters are removed, and subtype-representative k-mers are identified (step 4). Representative k-mers from these clusters are concatenated to form the dispensable and subtype-specific pangenome subsequences (step 5). The subtype-specific subsequences are then evaluated for their subtype mapping footprint by testing against the complete Influenza A database to eliminate false positives (step 6).

In the final phase (step 7), FM-indexes are created for the pangenome and tested on the full Influenza A database. The complete pangenome is used for general Influenza A identification, while subtype-specific subsequences from segments 4 and 6 are used for subtyping. Each step is detailed in the following subsections, outlining the methods and applications of this workflow.

#### 2.2.1 Input sequence preprocessing

The pangenome generation begins with a group of input sequences, denoted as G. Each sequence $S_{k}\in G$, where k= 1,2,3,…, n, and n is the total number of sequences, is divided into consecutive k-mers of length l=200 using a stride of r= 50 (step 1), resulting in a 150-nucleotide overlap (q) between adjacent k-mers (step 1). These parameters satisfy the condition r=l-q; the 150-nucleotide overlap corresponds to the most common read length in current high-throughput Illumina sequencing platforms for whole-genome and targeted sequencing applications (Vasiliadis *et al.* 2025, Wang *et al.* 2025). This ensures that each 150 bp read is fully contained within exactly one k-mer window, improving mapping and classification specificity.

For each sequence $S_{k},$ the set of k-mers, denoted as ${\mathrm{KMERS}}_{K}=\{{\mathrm{kmer}}_{k,1},{\mathrm{kmer}}_{k,2},\;{\mathrm{kmer}}_{k,3},\;\ldots ,\;{\mathrm{kmer}}_{k,m}\}$ is generated, where m, the total number of k-mers from sequence $S_{k},$ is calculated as:


(1) m=len(Sk)-1r+1

The complete set of k-mers from all sequences in G is denoted as:


(2) KMERT=∪k=1n {KMERSK | k= 1,2,…,n}

All k-mers containing unidentified or ambiguous nucleotides (e.g. “*N*”) were removed to ensure quality (step 2).

To assess the robustness of the method across different sequencing protocols, additional reduced databases were generated simulating 75 and 300 bp reads, which correspond to other standard read lengths in Illumina sequencing. These configurations used l = 125, r = 50 and l = 350, r = 50, respectively, also satisfying the condition r=l-q. However, the main results and benchmarking throughout the manuscript are based on the 150 bp configuration, reflecting its predominance in current sequencing practices.

#### 2.2.2 Clustering of k-mers

All k-mers from KMERT were clustered (step 3) using CD-HIT v.4.8.1 (Fu *et al.* 2012), with a sequence identity threshold of 89%. This value was selected to maintain subtype-specific clusters and ensure accurate classification. Given that aligners such as Bowtie2 (Langmead and Salzberg 2012) accept alignments with identities as low as 90%, higher clustering thresholds increased redundancy and, in some cases, caused reads to map to multiple clusters or subtypes. In contrast, lower thresholds occasionally merge unrelated k-mers, reducing subtype specificity and impairing alignment. To evaluate this threshold, values of 85% and 95% were evaluated during pipeline development (data not shown), confirming that with 95% some reads mapped to multiple clusters, while 85% occasionally caused reads not to be classified. Based on this clustering process, two types of clusters were defined:

- Multiple-element clusters (MEC), which are clusters containing multiple k-mers and a single representative k-mer, defined as:
  (3) MEC={C1,C2,…,Co | o>1}
  where o is the number of clusters with multiple k-mers gathered from KMERT.
- One-element clusters (OEC), which are clusters containing only one k-mer, where the k-mer is itself the representative sequence, defined as:
  (4) OEC={OC1,OC2,OC3,….OCv | v>1}
  where v is the number of single k-mer clusters derived from KMERT.

This clustering approach differentiates highly similar k-mers (MEC) from subtype-specific k-mers (OEC).

#### 2.2.3 Identification and reclassification of subtype-specific k-mers

Metadata of Influenza A subtypes is retrieved for each k-mer within every cluster to assess subtype specificity. Clusters classified as MEC were labeled as “dispensable” if their k-mers were associated with multiple Influenza A subtypes, reflecting high redundancy and reduced specificity. Conversely, k-mers from OEC clusters were labeled as “subtype-specific” because they represent unique regions with minimal similarity to other subtypes.

MEC  clusters with high taxonomic homogeneity, where over 95% of its k-mers are associated with the same Influenza A subtype, were reclassified as OEC clusters. This reclassification (step 4) recognizes their functional equivalence to OEC clusters and enhances their value for subtype-specific analyses.

#### 2.2.4 Construction of the pangenome by fusion of representative k-mers

Representative k-mers from MEC and OEC clusters were analyzed to reduce redundancy and construct longer subsequences. If two or more consecutive representative k-mers $\left({\mathrm{kmer}}_{k,i},\;{\mathrm{kmer}}_{k,i+1},\ldots ,\;{\mathrm{kmer}}_{k,i+n}\right)$ belong to the same Influenza A sequence ($S_{k}$) and the same cluster type (MEC or OEC), they were concatenated to form a single representative subsequence (step 5). This resulting pangenome is defined as:


(5) PANSEQ={PRP1,t, PRP2,t,.,PRPp,t}

where PRP represents subsequences derived from concatenated k-mers, p is the total number of subsequences, and t Indicates either the specific Influenza A subtype (for OEC-derived “subtype-specific” subsequences), or the label “dispensable” (for MEC-derived subsequences reflecting high redundancy).

This structured approach ensures that the pangenome captures subtype-specific regions with high specificity and redundant subsequences, improving the accuracy of subtype classification. For subtype-specific classification, segments 4 (HA) and 6 (NA) were analyzed and compressed independently. Each subtype-specific sequence was labeled according to its corresponding hemagglutinin (H) or neuraminidase (N) subtype (e.g. H3 or N2), allowing flexible detection of individual HA and NA components and facilitating the identification of novel subtype constellations. In contrast, the remaining six internal segments (PB2, PB1, PA, NP, M, and NS) were compressed using the full subtype annotation (e.g. H1N1 or H5N2). This strategy preserves the natural linkage between internal segment variability and its associated HA/NA combination, which is critical for tracking reassortment patterns and host-specific adaptations. Treating internal segments in this way enables the pangenome to capture segment-level diversity in the context of epidemiologically relevant subtypes, improving subtype-level resolution without fragmenting conserved segmental signatures.

#### 2.2.5 Refinement of subtype-specific pangenome subsequence

As the Influenza A virus dataset used to build PANSEQ was subsampled, it was necessary to validate that the subtype-specific subsequences in PANSEQ were truly subtype-specific by mapping the complete Influenza A virus database against them. The validation procedure (step 6) consisted of the following steps:

- Index creation: An FM-index was constructed for subtype-specific subsequences PRP∈PANSEQ.
- k-mer division: Sequences from the complete Influenza A database were divided into k-mers of 150 bp length with a stride of 50. Metadata assigned the subtype origin for each k-mer.
- Mapping: The k-mers were mapped using Bowtie2 v.2.3.4 (Langmead and Salzberg 2012), with the—very-sensitive option and -k 100 to retrieve up to 100 best-scoring alignments per k-mer. The FM-index used was generated using Bowtie2-build with default parameters.
- Subtype mapping footprint (SMF): For each subtype-specific subsequence ${\mathrm{PRP}}_{kt}$, its subtype specificity was quantified using the formula:
  (6) SMFk, t =stypehits
  where k is the index of ${\mathrm{PRP}}_{k,t}$, t is the Influenza A subtype, hits represents the total number of k-mers matches for ${\mathrm{PRP}}_{k,t}$ and stype is the number of matches belonging to subtype t. Subtype specificity was quantified using the SMF, defined as the proportion of k-mer matches belonging to a given subtype.
- Classification: A subsequence ${\mathrm{PRP}}_{k,t}$ was retained as a subtype-specific pangenomic subsequence If ${\mathrm{SMF}}_{k,t\;}>0.95$. Otherwise, it was relabeled as dispensable.

All steps in the pangenome generation workflow were executed using eight computational threads to ensure consistent and reproducible performance.

### 2.3 Evaluation metrics

#### 2.3.1 Relative compression index

The Relative Compression Index (RCI) quantifies the effectiveness of the pangenomes in compressing the information from the original dataset. It is calculated as:


(7) RCI=1-∑k=1p len(PRPk)∑u=1n len(sequ)

where, ${\mathrm{PRP}}_{k}$ represents the *k*th subsequence in PANSEQ (complete or subtype-specific), ${\mathrm{seq}}_{u}$ denotes *u*th sequence from the original dataset, len is the length in nucleotides, p and n are the total number of subsequences in PANSEQ and sequences in the original database, respectively.

The RCI reflects the proportion of the original dataset represented by the pangenome subsequences. Higher RCI values indicate a more effective compression, demonstrating the pangenome’s ability to retain essential information with fewer subsequences.

#### 2.3.2 Recovery index and general metrics

The recovery index (RI) evaluates the pangenome’s ability to map sequences from a test dataset. It is calculated:


(8) RI=hits|STEST|

where, hits is the number of test sequences with significant matches to PANSEQ, and | STEST | represents the total number sequences in the test dataset.

The RI indicates the proportion of test sequences successfully mapped to the pangenome. A higher RI indicates that the pangenome effectively captures the diversity of the test sequences, while a lower RI implies the underrepresentation of certain regions or sequences.

To evaluate subtyping, the following metrics are used:


(9) precision=TPTP + FP

(10) True Positive Rate (TPR)=TPTP + FN 

(11) False Negative Rate (FNR)=FNTP + FN 

where TP represents true-positive predictions for a given class, FP are false-positive predictions and FN are false-negative predictions.

TPR measures the proportion of actual positives correctly identified, while the FNR reflects the proportion of positives missed by the pangenome.

### 2.4 Evaluation of pangenome performance

The performance of the complete and subtype-specific pangenomes was evaluated using two independent test datasets: one consisting of newly annotated Influenza A sequences from public databases, and the other comprising real sequencing data from PCR-confirmed clinical samples.

#### 2.4.1 *In silico* validation using newly annotated NCBI sequences

This first test dataset consists of recently annotated Influenza A virus sequences downloaded from NCBI between 9 May 2022, and 28 May 2024. These sequences were not part of the original pangenome construction, which used sequences released prior to 8 May 2022. This temporal separation ensured that the evaluation was conducted on independent and previously unseen data, enabling an unbiased assessment of the pangenome’s ability to recover and classify novel Influenza A sequences.

To carry out this evaluation, sequences annotated under taxon ID “11320” (corresponding to *Alphainfluenzavirus*) were used to test the complete pangenome, while sequences labeled as “102796” (H9N2 subtype) and “102793” (H5N1 subtype) were selected to evaluate the subtype-specific pangenome (Table 2).

**Table 2.** Summary of the test dataset with initial sequences, valid k-mers, and nucleotide counts for Influenza A, and subtypes H5N1 and H9N2, which were released after the K-FluDB construction, enabling an unbiased evaluation.

Database	Initial sequences	Valid k-mers	nt^a^
Inf A	261 104	7 903 883	1 185 582 450
H5N1 subtype	21 333	647 036	97 055 400
H9N2 subtype	4151	126 227	18 934 050

a nt, nucleotide count.

The testing procedure consisted of four main steps:

- K-mer generation and pangenome indexing: Test sequences were divided into k-mers of 150 nucleotides with a stride of 50 nucleotides, simulating real sequencing reads, then the complete and subtype-specific pangenomes were indexed using Bowtie 2-build.
- Mapping to the subtype-specific pangenome: k-mers labeled as H5N1 and H9N2 were mapped to this index using Bowtie2 v2.3.4 with the—very-sensitive preset. The mapping results were analyzed (Table 2).
- Mapping to the complete pangenome: k-mers labeled as “11320” were mapped, and the percentage of k-mers identified as influenza A was calculated
- Evaluation: The RI was calculated for sequences mapped to both the complete and subtype-specific pangenomes, reflecting the proportion of successfully mapped test sequences. TPR and FPR were calculated for the subtype-specific pangenome to evaluate the accuracy of subtype typification.

This testing framework assessed the pangenome’s effectiveness in recovering and classifying new influenza sequences, providing insights into its applicability for emerging datasets.

#### 2.4.2 Identification of documented reassortant virus

To evaluate the capacity of K-FluDB to detect reassortant events, two previously reported Influenza A genomes with documented reassortment patterns were selected. These include one H3N8 virus from the study by Bao *et al.* (2022) and one H3N3 virus from He *et al.* (2024). Both genomes were processed using the same k-mer-based classification pipeline described above, and each segment was analyzed independently against the subtype-specific pangenomes to identify divergent subtype signatures.

#### 2.4.3 Experimental validation using clinical sequencing data

To complement the *in silico* evaluation, the pangenome was validated using real sequencing data derived from PCR-confirmed Influenza A cases. The samples and associated metadata were collected as part of Mexico’s national public health response to respiratory viruses. This effort was coordinated by the CoViGen-Mex consortium under the Official Mexican Standard NOM-017-SSA2-2012 for the epidemiological surveillance program. All samples were anonymized prior to analysis.

Oropharyngeal and nasopharyngeal swabs were obtained from hospitals and clinics of the Mexican Social Security Institute (IMSS), a member of the consortium. A total of 18 PCR-confirmed Influenza A samples, classified by subtype, formed the basis of this analysis. The samples were processed using the Illumina COVIDSeq kit (Illumina, San Diego, CA, USA) with primers specifically adapted for Influenza A, following the manufacturer’s protocol. Sequencing was performed on the Illumina NextSeq 550 platform with 2 × 150 paired-end runs, and basecalling was conducted using BCL Convert to generate FASTQ files.

Raw reads were preprocessed with Fastp 0.19.4 (Chen *et al.* 2018) to improve overall read quality by trimming low-quality bases and removing short reads, using the following non-default parameters:—cut_tail (for trimming low-quality bases at the 3′ end) and -l 40 (to discard reads shorter than 40 bp). Duplicate reads were subsequently removed using with CD-hit-dup v.4.8.1 (Fu *et al.* 2012), with default parameters. These sequencing data are available in Zenodo and can be accessed at https://zenodo.org/records/17203072. The preprocessed reads were mapped to the subtype-specific and complete pangenomes using Bowtie 2 v2.3.4 with the—very-sensitive preset. Then, the same evaluation procedures applied to newly annotated NCBI sequences were also implemented to ensure consistency. All steps in this test workflow were also executed using eight computational threads.

This well-characterized dataset provided a robust foundation for validating the pangenomes, enabling the assessment of their recovery index (RI) and true positive rate (TPR). This experimental validation demonstrated the applicability and robustness of the pangenomes in detecting and accurately classifying Influenza A viruses in real-world genomic datasets.

### 2.5 Comparison with existing databases and tools

To evaluate the performance and distinct features of K-FluDB, three comparative analyses were performed: (i) accuracy in subtype identification, (ii) reduction of database redundancy, and (iii) coverage of subtype diversity across reference datasets. In all cases, the complete Influenza A dataset described in Section 2.1 was used, representing 48 distinct subtypes, comprising xx HA and yy NA. For this purpose, sequences were processed into k-mers using 150 bp windows with a xx bp step size.

For subtype classification, K-FluDB was compared against INSaFLU, a widely used platform for influenza genomic surveillance and RefSeq database. Simulated reads were mapped using Bowtie2 against both the k-mer-based pangenome reference from K-FluDB and RefSeq. For INSaFLU, the online tool was used. Subtype predictions were derived from segment-level classifications based on the HA and NA segments, and accuracy was defined as the proportion of samples with all segments correctly assigned to the expected subtype.

To assess redundancy, K-FluDB was compared with the complete set of annotated Influenza A virus sequences available in GenBank. The reduction in database size was quantified by comparing the total number of bases in the k-mer-derived pangenome against the full GenBank dataset, calculated separately for each genomic segment.

## 3 Results and discussion

### 3.1 Global compression results

The complete pangenome comprises both dispensable and subtype-specific subsequences. Its primary function is to enable Influenza A identification without specific subtype classification. In contrast, the subtype-specific pangenome contains only subtype-specific sequences derived from segments 4 (hemagglutinin, H) and 6 (neuraminidase, N), allowing for precise mapping against Influenza A subtypes.

As summarized in Table 3, the complete pangenome achieved a 96.64% compression relative to the original database, while the subtype-specific pangenome attained an impressive 98.33% compression. Notably, despite this high level of compression, over 99.51% of the sequences in the original dataset could still be accurately recovered. These results highlight the elimination of genetic redundancy in the Influenza A genome while preserving essential information for subtype classification, underscoring the efficiency and utility of the generated pangenomes.

**Table 3.** Summary of K-FluDB pangenomes for 75, 150, and 300 bp reads, including the number of sequences and nucleotides per segment in the dispensable and subtype-specific components.

Pangenome type		Seqs^a^	nt^b^	RCI^c^
Input database		81 262	131 316 685	–
75 bp reads	Complete	22 765	4 373 590	96.67%
	Subtype-specific	10 008	2 273 708	98.27%
150 bp reads	Complete	15 373	4 412 173	96.64%
	Subtype-specific	6705	2 196 255	98.33%
250 bp reads	Complete	8273	3 788 073	97.11%
	Subtype-specific	3592	1 828 642	98.60%

a Seqs, number of sequences.

b nt, nucleotide count.

c RCI, relative compression index, representing the percentage of nucleotide reduction relative to input data.

The subtype-specific pangenome retained all 18 Hx and 11 Nx subtypes, successfully identifying every HxNx combination (Table 3). This confirms its capacity to capture segment-level diversity and to preserve the full range of subtype constellations, which is particularly relevant for identifying reassortment events. The high compression observed, especially in the subtype-specific datasets, reflects the robust design of the database, which minimizes redundancy while maintaining essential subtype-specific information.

**Figure 2.**
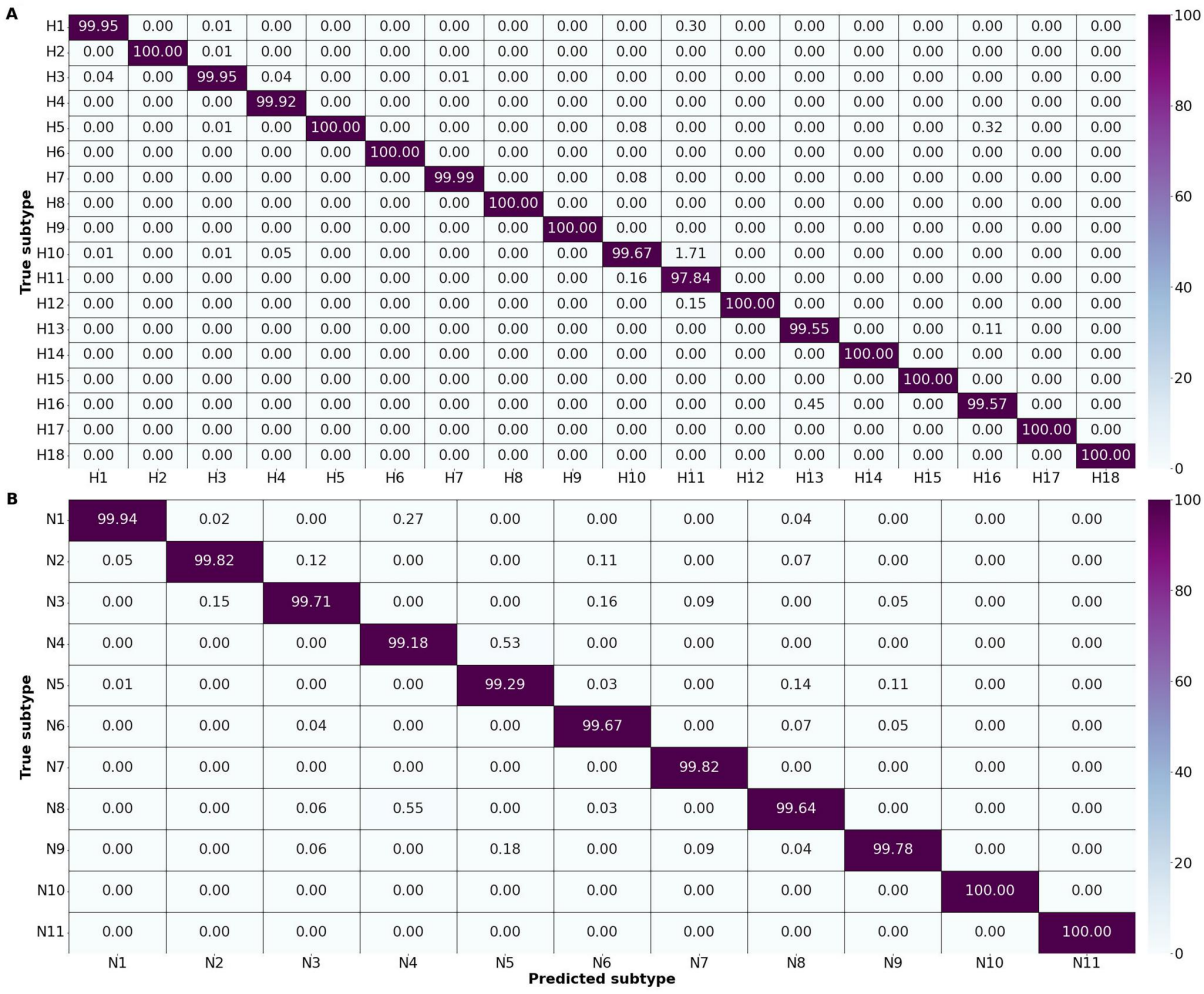
Confusion matrices showing TPR and FPR for subtype-specific sequence classification. (A) segment 4 (H-hemagglutinin). (B) segment 6 (N-neuraminidase). High-diagonal TPR indicates accurate subtype classifications, while off-diagonal FPR reflects rare misclassifications, primarily among closely related subtypes.

**Figure 3.**
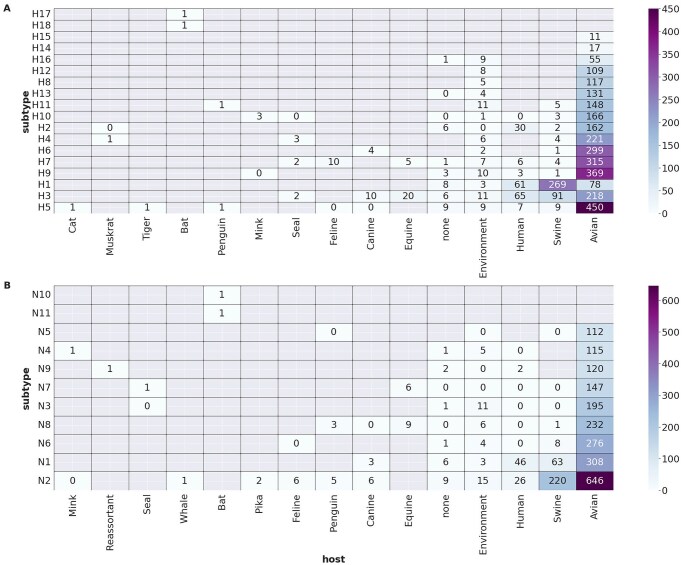
Heatmap of existing subtypes in subtype-specific sequences of the influenza A pangenome, stratified by host; the total amount of subtypes is 50. (A) Segment 4 (H). (B) Segment 6 (N).

Figure 2 evaluates the classification performance in terms of TPR and False Positive Rate (FPR) for the 18 hemagglutinin (H) and 11 neuraminidase (N) subtypes. Panel (A) focuses on the H subtypes, while Panel (B) illustrates the N subtypes, both exhibiting near-perfect diagonal alignments that highlight the model’s high accuracy. For Hx subtypes, the average precision and TPR reach 98.8% and 99.77%, respectively, as detailed in Table 1, available as supplementary data at *Bioinformatics Advances* online. Similarly, for Nx subtypes, the average precision and TPR are 99.71% and 99.4%, respectively, as shown in Table 2, available as supplementary data at *Bioinformatics Advances* online. The detailed confusion matrices in Tables 3 and 4, available as supplementary data at *Bioinformatics Advances* online, present the number of sequences for each class prediction. Subtypes H13 (99.55%), H16 (99.57%), H10 (99.67%), H11 (97.84%), N4 (99.18%), N5 (99.29%), and N8 (99.64%) showed slightly lower TPRs, possibly due to their limited representation and avian host restriction (Fig. 3). However, other subtypes with similar profiles, such as H4, H5, and H7, achieved near-perfect classification. These results suggest no clear pattern underlying the few misclassifications observed, supporting the overall robustness of the model.

**Table 4.** Summary of k-mer reduction in the influenza A database, showing the distribution of sequences and nucleotides across segments. Percentages reflect the proportional size of the reduced database relative to the original.

Segment			1 (PB2)	2 (PB1)	3 (PA)	4 (HA)	5 (NP)	6 (NA)	7 (M1, M2)	8 (NS1, NEP)
Initial seqs			9811	9946	9868	11 287	9872	10 478	10 056	9944
Initial nt			21 962 041	22 239 834	21 121 06	18 222 760	14 841 330	14 547 376	9 902 780	8 479 502
75 bp reads^a^	Disp.^b^	seqs^a^	3474	2818	2840	103	1839	116	720	847
		nt^e^	574 550 (2.62%)	447 300 (2.01%)	451 200 (2.14%)	27 325 (0.15%)	309 275 (2.08%)	24 500 (0.17%)	112 150 (1.13%)	140 825 (1.66%)
	Sub spec.^c^	seqs^d^	219	141	144	5316	137	3895	58	98
		nt^e^	55 875 (0.25%)	34 275 (0.15%)	40 050 (0.19%)	1 237 450 (6.79%)	30 475 (0.21%)	836 375 (5.75%)	11 650 (0.12%)	17 550 (0.21%)
150 bp reads^a^	Disp.^b^	seqs^d^	2312	1978	1920	71	1300	62	477	546
		nt^e^	603 350 (2.75%)	487 050 (2.19%)	484 700 (2.29%)	26 000 (0.14%)	330 950 (2.23%)	19 050 (0.13%)	114 700 (1.16%)	139 400 (1.64%)
	Sub spec.^c^	seqs^d^	146	87	72	3613	64	2627	36	60
		nt^e^	53 850 (0.25%)	31 600 (0.14%)	32 650 (0.15%)	1 204 350 (6.61%)	20 400 (0.14%)	820 000 (5.64%)	10 600 (0.11%)	16 100 (0.19%)
300 bp reads^a^	Disp.^b^	seqs^d^	1235	1197	978	41	683	35	245	267
		nt^e^	531 600 (2.42%)	482 850 (2.17%)	409 750 (1.94%)	21 300 (0.12%)	284 650 (1.92%)	16 250 (0.11%)	97 350 (0.98%)	111 000 (1.31%)
	Sub spec.^c^	seqs^d^	67	49	32	1946	29	1432	16	21
		nt^e^	36 900 (0.17%)	29 100 (0.13%)	26 100 (0.12%)	1 006 950 (5.53%)	14 450 (0.10%)	693 100 (4.76%)	8250 (0.08%)	10 200 (0.12%)

a bp, base pairs.

b Disp, dispensable, refers to conserved sequences shared among subtypes.

c Sub spec, subtype-specific, indicates sequences unique to a given subtype.

d seqs, number of sequences.

e nt, nucleotide count.

### 3.2 Segment-level compression analysis

The segment-level compression results of all Influenza A subtypes revealed the full spectrum of genetic diversity across the virus’s eight genomic segments (Table 4), with compression times detailed in Table 5, available as supplementary data at *Bioinformatics Advances* online. Segments 4 and 6 exhibited the highest variability, reflecting their critical roles in viral infectivity and immune evasion, as they are key antigenic targets of the adaptive immune system (Bhatt *et al.* 2011, Poon 2024). Segment 4, encoding hemagglutinin (HA), showed 0.14% dispensable and 6.61% subtype-specific sequences, consistent with HA’s role in host cell binding and immune evasion, highlighting its adaptation to neutralizing antibodies. Similarly, segment 6, encoding neuraminidase (NA), displayed 0.13% dispensable and 5.64% subtype-specific sequences. This diversity reflects adaptation to selective pressures, including neuraminidase inhibitors like oseltamivir.

**Table 5.** Mapping results for newly gathered sequences for H5N1 and H9N2 sequences against the K-FluDB complete and subtype-specific pangenomes, including subtypes prediction.

Test database	Initial sequences		Recovery index (RI%)^a^		Hx type	Nx type
	Sequences	k-mers	Complete	Sub spec.	k-mers mapped (TPR%)^b^	k-mers mapped (TPR)
Inf A	261 104	7 903 883	7 865 521 (99.51%)	2 017 726 (25.52%)	–	–
H5N1	21 333	647 036	645 027 (99.69%)	168 502 (26.04%)	H5, 98 595 (100.0 )	N1. 69 907 (100.00%)
H9N2	4151	126 227	121 539 (96.28%)	34 788 (27.56%)	H9, 523 005 (97.52%)	N2, 11 199 (100.00%)

a RI, recovery index.

b TPR, true positive rate.

Segments 1 (PB2), 2 (PB1), 3 (PA), and 5 (NP) displayed intermediate variability, with dispensable sequence proportions ranging from 2.19% to 2.75%. Their relatively large size may partially explain the higher number of dispensable k-mers detected. However, other factors likely contribute as well, including their functional roles in the viral polymerase complex and ribonucleoprotein assembly, as well as selective pressures linked to host adaptation. Segment 1 showed 2.75% dispensable sequences, reflecting its essential role in host specificity and zoonotic transmission. Segment 2 (PB1) exhibited 2.19%, with variability partly due to the embedded PB1-F2 open reading frame associated with virulence. Segment 3 (PA) displayed 2.29%, highlighting its role in RNA synthesis and polymerase interactions. Segment 5 (NP), with 2.23%, is subject to immune-driven selection due to its role in RNA encapsulations.

In relation to the compression strategy designed for 150-nucleotide reads, segments 7 (M1, M2) and 8 (NS1, NEP) were the least variable, exhibiting the highest compression ratios, with 1.16% and 1.64% of rest information from the original output, respectively (Table 4). In addition to their critical structural and regulatory roles, their short genomic length likely limits the number of k-mers that can vary across subtypes. Segment 7 encodes matrix proteins essential for viral assembly and ion channel activity, while segment 8 encodes proteins involved in replication control and host immune modulation. The minimal variability of these segments aligns with their critical functions and strong evolutionary constraints combined with reduced sequence length for divergence. Compression based on representative k-mer clustering revealed that shorter k-mer lengths resulted in lower compression rates across all segments. For instance, in segments 4 and 6, using 75-nt k-mers produced subtype-specific pangenome fragments representing 6.75% and 5.75% of the original data, respectively. In contrast, increasing the k-mer size to 300 nucleotides reduced these values to 5.53% and 4.76%. This trend was consistent across segments and subtypes. The higher compression observed with longer k-mers is likely due to their ability to capture broader sequence context; since clustering is performed within the same viral species, longer k-mers are more likely to span conserved regions, facilitating the identification and collapse of redundant sequences.

#### 3.2.1 Limitations and interpretative considerations

Globally, the subtype classification and identification of Influenza A viruses rely exclusively on the hemagglutinin (H) and neuraminidase (N) genes, introducing biases that obscure the variability and evolutionary dynamics of other genomic segments (Beniston and Skittrall 2024). This approach indirectly infers the evolution of non-surface glycoprotein segments (e.g. PB2, PB1, PA, NP, M1/M2, NS1/NEP), limiting insights into their independent evolution and adaptations. Additionally, the current system overlooks reassortment events, which are frequent in Influenza A and allow genomic segments to originate from distinct lineages (Lin *et al.* 2023). As a result, variability in other segments often reflects their association with specific H and N subtypes rather than their own evolutionary trajectories.

Moreover, differences in segment size further complicate these analyses, as larger segments (e.g. PB2, PB1, and PA) inherently contain more sequence content for compression, which may inflate the proportion of dispensable sequences relative to smaller segments like M1/M2 or NS1/NEP. This size-related bias can obscure true variability and lead to misinterpretation of the evolutionary constraints acting on each segment.

To overcome these limitations, future studies should include independent phylogenetic analyses of individual segments and normalize for segment size to reduce compression-related biases. Such efforts would provide a more comprehensive understanding of the evolutionary pressures acting on the entire Influenza A genome, including reassortment events, host-specific adaptations, and the potential for segment-specific functional innovations.

### 3.3 Host-specific distribution and subtype analysis

Analysis of Influenza A subtypes in segments 4 and 6 highlights distinct host-specific patterns and subtype distributions. The dataset comprises sequences from avian species, swine, humans, and other mammals such as dogs, cats, and seals. Avian, human, and swine hosts dominate, reflecting the zoonotic nature of influenza viruses. Avian species, as the largest reservoir, host the broadest subtype diversity, playing a pivotal role in influenza ecology and evolution. Swine, recognized as mixing vessels for human and avian strains, shows notable diversity, while humans are primarily linked to seasonal subtypes.

Subtype diversity varies significantly by host. H1N1 and H3N2 dominate in humans and swine, reflecting their established circulation and zoonotic potential. These subtypes are central to pandemic and seasonal epidemics, emphasizing their public health relevance. In avian hosts, subtypes H5, H6, and H7, including H5N1, H5N2, H6N1, H6N2, and H7N9, are prominent and historically associated with avian-origin outbreaks. H5N1 stands out for its prevalence and cross-species transmission, posing significant zoonotic risks. Additionally, H9N2, abundant in avian hosts, is occasionally detected in swine and humans, suggesting sporadic cross-species transmission and potential as a reassortment source. The environmental category also contributes to subtype diversity, with subtypes such as H3N6 and H6N6 being well represented. This indicates that environmental reservoirs or contamination may facilitate subtype maintenance and interspecies transmission.

The k-mer-based sequence reduction process across the eight segments effectively filters out less significant host-subtype combinations, retaining those with the highest epidemiological relevance (Fig. 3). Prominent subtype-host combinations, such as H5N1 in avians, H1N2 in swine, and H3N2 in humans, remain well represented, reflecting their importance in surveillance and distinct genetic markers. Less common combinations, such as H7N7 in certain mammals, are filtered out, likely due to high sequence similarity or limited genetic distinctiveness, enabling a more focused analysis on critical subtypes relevant for monitoring outbreaks.

Segments 4 and 6 are particularly significant due to their role in subtype typing, frequently used in PCR-based studies to identify circulating subtypes. Both segments retain a dense representation of key subtypes across diverse hosts, crucial for effective surveillance. For instance, H7N9 remains predominantly associated with avian hosts in segment 4, as shorter sequences derived from human host are filtered out, reinforcing its link to avian reservoirs. Similarly, H1N2 is retained exclusively in swine, underscoring its host specificity and the method’s ability to capture essential subtype-host interactions, even with incomplete data.

The results highlight the utility of k-mer-based reduction for prioritizing subtype-host combinations with high epidemiological significance, while segments 4 and 6 provide robust markers for subtype identification and monitoring.

### 3.4 Pangenome evaluation results

The performance of the pangenome was evaluated using the two independent test datasets described in Section 2.4. The newly annotated sequences from NCBI and the real genomic data from clinical samples.

#### 3.4.1 *In silico* validation with newly annotated sequences

A set of newly annotated Influenza A sequences (Table 5) was processed to generate 7 903 883 k-mers, which were then mapped against the complete pangenome. Of these, 7 865 521 were correctly identified as Influenza A, resulting in a recovery index of 99.51%, indicative of a high level of similarity between the new sequences and the pangenome.

Subtype-specific mapping results for H5N1 and H9N2 are also shown in Table 5. For H5N1, the recovery index was 99.69% against the complete pangenome, while H9N2 achieved 96.29%. In contrast, when mapping k-mers against the subtype-specific pangenome, the recovery indices dropped to 26.04% for H5N1 and 27.56% for H9N2, reflecting the higher stringency of this dataset, which retains only unique HA and NA sequences.

#### 3.4.2 Validation with documented reassortant genomes

To further evaluate the ability of K-FluDB to detect reassortment events, two genomes with previously documented reassortant origins were analyzed. For the H3N8 virus described by Bao *et al.* (2022), segments 2, 3, and 4 showed k-mer matches to H3N2, segment 6 to N8, and segment 7 to H9N2. Additionally, segment 8 presented matches to both H3-N8 and H9-N2 subtype combinations (Table 6). These findings are consistent with the reassortment pattern reported in the original study, which described the virus as a reassortant containing segments derived from H3N2, H9N2, and other avian sources.

**Table 6.** Segment-level subtype predictions for two documented reassortant Influenza A viruses, showing the number of matching k-mers and the assigned subtype for each segment.

Segment	Bao *et al.* (2022)		He *et al.* (2024)	
	k-mers	Found subtypes	k-mers	Found subtypes
1 (PB)	–	–	–	–
2 (PB1)	1, 1	H3-N2	1, 1	–
3 (PA)	1	H3-N2	1	H3-N2
4 (HA)	5	H3	5	H3
5 (NP)	–	–	–	–
6 (NA)	13	N8	9	N3
7 (M1, M2)	2	H9_N2	4	H9-N2
8 (NS1, NEP)	1, 1	H3-N8, H9_N2	2, 2	H3-N8, H9-N2

Similarly, the H3N3 virus described by He *et al.* (2024) showed segment-specific subtype assignments matching the reported reassortant structure. K-FluDB identified segments 3 and 4 as H3, segment 6 as N3, and segment 7 as H9N2, with segment 8 again showing mixed subtype signatures (Table 6). This constellation agrees with the triple-reassortant nature described in the publication, confirming that K-FluDB effectively supports the identification of intra-genomic inconsistencies in line with known reassortment events.

#### 3.4.3 Experimental validation with clinical sequencing data

The complete and subtype-specific pangenomes were also evaluated using sequencing data from 18 PCR-confirmed Influenza A clinical samples (Table 7). Samples 1–9 were identified as H1N1, and samples 10–18 as H3N2. Mapping against the complete pangenome was used to identify Influenza A without subtype annotation, while mapping against the subtype-specific pangenome served as a subtyping predictor.

**Table 7.** Mapping results for 18 genomic samples against the K-FluDB complete and subtype-specific pangenomes, including subtype predictions.

Sample Id	Reads	PCR type	K-mers mapped to K-FluDB (RI%)^a^		Hx subtype	Nx subtype
			Complete	Subtype-specific	K-mers mapped (TPR%)^b^	K-mers mapped (TPR%)
01020866UIMY	144 500	H1N1	117 173 (81.09%)	11 514 (7.97%)	H1, 5,439 (99.32%)	N1, 6,032 (99.90%)
01021509UIMY	225 222	H1N1	209 628 (93.08%)	27 805 (12.35%)	H1, 9,447 (99.17%)	N1, 18,263 (99.91%)
01021641UIMY	264 098	H1N1	242 483 (91.82%)	44 971 (17.03%)	H1, 17,284 (99.60%)	N1, 27,611 (99.97%)
01021651UIMY	278 589	H1N1	266 290 (95.59%)	47 879 (17.19%)	H1, 18,242 (99.45%)	N1, 29,527 (99.97%)
01021863UIMY	222 012	H1N1	166 351 (74.93%)	15 756 (7.10%)	H1, 5,100 (99.53%)	N1, 10 624 (99.92%)
01021899UIMY	258 869	H1N1	240 944 (93.08%)	44 805 (17.31%)	H1, 14 187 (99.27%)	N1, 30 501 (99.96%)
01022143UIMY	259 288	H1N1	241 323 (93.07%)	34 246 (13.21%)	H1, 10 955 (99.48%)	N1, 23 227 (99.97%)
01024183UIMY	124 584	H1N1	116 286 (93.34%)	20 186 (16.20%)	H1, 8180 (99.71%)	N1, 11 974 (99.93%)
01024536UIMY	332 814	H1N1	315 018 (94.65%)	56 980 (17.12%)	H1, 19 626 (99.41%)	N1, 37 218 (99.95%)
01000123UIMY	144 024	H3N2	133 153 (92.45%)	24 030 (16.68%)	H3, 9608 (100%)	N2, 14 422 (100%)
01006970UIBMZ	152 089	H3N2	138 084 (90.79%)	24 470 (16.09%)	H3, 9705 (100%)	N2, 14 765 (100%)
01006974UIBMZ	178 142	H3N2	171 435 (96.24%)	28 087 (15.77%)	H3, 10 003 (100%)	N2, 18 084 (100%)
01006991UIBMZ	144 521	H3N2	129 424 (89.55%)	18 699 (12.94%)	H3, 6985 (100%)	N2, 11 714 (100%)
01009323UIBMZ	190 406	H3N2	179 690 (94.37%)	33 994 (17.85%)	H3, 13 312 (100%)	N2, 20 682 (100%)
01017109UIMY	223 361	H3N2	191 684 (85.82%)	27 533 (12.33%)	H3, 10 794 (100%)	N2, 16 739 (100%)
01040096CIBIN	441 202	H3N2	406 680 (92.18%)	60 308 (13.67%)	H3, 22 534 (100%)	N2, 37 774 (100%)
01113643LCE	175 407	H3N2	158 535 (90.38%)	24 505 (13.97%)	H3, 8139 (99.99%)	N2, 16 365 (100%)
01122196LCE	378 982	H3N2	342 136 (90.28%)	38 227 (10.09%)	H3, 16 212 (99.99%)	N2, 22 013 (100%)

a RI, recovery index.

b TPR, true positive rate.

Mapping reads to the complete pangenome ranged from 74.93% to 96.24%, reflecting the sample quality in terms of Influenza A specificity, as the samples contained genomic information from multiple organisms. Mapping reads to the subtype-specific pangenome were lower, ranging from 7.10% to 17.85%, as this pangenome contains only subtype-specific genomic regions.

When analyzed independently, accuracy for segment 4 and segment 6 improved significantly, ranging from 99.17% to 100% for segment 4 and 99.90% to 100% for segment 6. These results highlight the effectiveness of segment-specific mapping for accurate subtype predictions in genomic samples.

### 3.5 Comparative results of K-FluDB, RefSeq, and INSaFLU

The performance of K-FluDB was assessed against RefSeq and INSaFLU (Borges *et al.* 2018) using simulated reads mapped to the subtype-specific pangenomes of segments 4 (HA) and 6 (NA). K-FluDB demonstrated higher sensitivity and subtype resolution across both segments (Tables 8 and 9), achieving mapping rates of 95.5% to 100% for HA (Table 8) and 92.31% to 100% for NA (Table 9) when compared to the original subtype annotations. In contrast, RefSeq, due to its compact and non-redundant design, showed poor recovery even for correctly annotated cases, reflecting a loss of subtype diversity. INSaFLU, while capable of detecting more subtypes, does not quantify mapping performance and lacks resolution for identifying annotation errors or reassortants due to its contig-based approach.

**Table 8.** Mapping results of simulated HA reads against RefSeq and K-FluDB subtype-specific pangenomes.

Original subtype	Seqs	k-mers	k-mers mapped		Subtype identified		
			Refseq	Pangenome subtype-specific	Refseq	Pangenome	INSaFLU
H1	40 394	560 759	363 804 (64.9%)	55 0260 (98.1%)	H1, H3	H1, H3, H11	H1
H2	702	10 678	2514 (23.5%)	10 442 (97.8%)	H2	H2, H3	H2, H3
H3	40 258	538 189	404 961 (75.3%)	525 758 (97.7%)	H3, H1, H5	H3, H1, H4, H7, H5	H3, H1
H4	2118	33 021	0	32 632 8 (98.8%)	_	H4	H4
H5	6834	101 550	60 776 (59.9%)	96 999 (95.5%)	H5, H1	H5, H3, H10, H16, H1	H5
H6	1930	29 541	0	28 580 (96.8%)	–	H6	H6
H7	3245	50 672	25 286 (49.9%)	49 707 (98.1%)	H7	H7, H10, H3	H7
H8	188	2864	0	2835 (99.0%)	–	H8	–
H9	4337	65 355	13 174 (20.2%)	63 326 (97.9%)	–	H9	H9
H10	1269	19 794	0	19 338 (97.7%)	–	H10, H11, H3, H1, H4	H10, H11
H11	679	10 650	0	10 202 (95.8%)	–	H11, H10	H11
H12	233	3673	0	3618 (98.8%)	–	H12, H11	H12
H13	525	8500	0	8144 (95.8%)	–	H13, H3, H16	H13
H14	28	447	0	445 (99.8%)	–	H14	H14
H15	20	330	0	329 (99.7%)	–	H15	–
H16	275	4472	0	4334 (96.9%)	–	H16, H13	H16, H13
H17	3	51	0	50 (98.0%)	–	H17	–
H18	2	34	0	34 (100%)	–	H18	–

**Table 9.** Mapping results of simulated NA reads against RefSeq and K-FluDB subtype-specific pangenomes, including subtypes predicted by each tool and additional K-FluDB predictions validated by BLAST.

Original subtype	Seqs^a^	k-mers	k-mers mapped		Identified Subtype		
			Refseq	Pangenome	Refseq	Pangenome	INSaFLU
N1	30 870	382 408	252 511 (66.03%)	369 604 (96.65%)	N1, N2	N1, N2, N4, N8	N1, N4
N2	30 427	386 313	291 344 (75.42%)	368 182 (95.31%)	N2, N1	N2, N1, N6, N8, N3	N2, N1
N3	1624	21 103	594 (2.81%)	205 78 (97.51%)	N2, N9	N3, N2, N6, N9, N7	N3, N2
N4	371	4775	0	4688 (98.18%)	–	N4, N5, N2	N4
N5	576	7435	4 (0.05%)	7260 (97.65%)	N9	N5, N8, N9, N1, N6, N2	N5, N8
N6	2912	37 717	1 (0.00%)	36 738 (97.40%)	N9	N6, N8, N9, N3	N6
N7	1115	14 202	0	13 980 (98.44%)	–	N7	N7
N8	2806	36 453	0	35 116 (96.33%)	–	N8, N4, N6, N3	N8
N9	1886	24 056	16 740 (69.59%)	23 512 (97.74%)	N9	N9, N8, N7, N5, N3	N9
N10	3	39	0	36 (92.31%)	–	N10	–
N11	2	26	0	26 (100%)	–	N11	–

a seqs, number of sequences.

K-FluDB also recovered additional HA or NA subtypes in several cases. For instance, avian-derived sequences originally labeled as H1 showed exclusive matches to H10-specific k-mers, supported by BLAST results, revealing wrong annotations. These patterns were more frequent in avian sequences, consistent with the broader subtype diversity observed in this host group (Fig. 3).

In addition, multiple subtype signals were detected across distinct segments in some genomes, suggesting reassortment or coinfection. By preserving subtype-specific k-mer profiles for all eight segments, K-FluDB enables identification of discordant constellations not captured by methods dependent on fixed subtype pairings.

### 3.6 Practical implementation and future developments

K-FluDB can be used directly as a reference database in genomic surveillance pipelines, using standard aligners such as Bowtie2, to enable rapid detection, subtyping, and reassortment analysis of Influenza A viruses from high-throughput sequencing data. Its structure, based on subtype-specific and dispensable k-mer subsequences, supports accurate segment-level classification and facilitates the selection of optimal reference genomes for downstream applications, including consensus sequence reconstruction and phylogenetic analysis.

To improve accessibility for users without command-line experience, K-FluDB is compatible with integration into user-friendly platforms such as Galaxy and INSaFLU. A web-based interface is also under development, which will allow data upload, automated subtype prediction, reassortment visualization, and genome sequence generation.

The complete pipeline, from sequence retrieval to pangenome construction, can be executed in ∼2 hours using eight processing threads, allowing efficient regeneration. K-FluDB will be updated every 6 months to incorporate newly available Influenza A sequences and to generate versions adapted to common read lengths (75, 150, and 300 nucleotides). Updated releases will be published on Zenodo with version control and changelogs and integrated into the web interface. The database and associated tools will be maintained and updated for at least 5 years following publication to ensure long-term utility in genomic surveillance.

## 4 Conclusions

K-FluDB represents a pivotal advancement in the genomic surveillance of Influenza A viruses, addressing challenges such as redundant reference databases and the need for high-resolution subtype differentiation. Through a k-mer-based approach, K-FluDB achieves significant compression without compromising essential genomic information, offering a streamlined yet comprehensive resource for identifying Influenza A subtypes.

One of K-FluDB’s most noteworthy contributions is its ability to assist the detection of reassortment events, a key driver of viral evolution and pandemic potential. By accurately identifying multiple subtypes within a single segment, K-FluDB enhances our understanding of the mechanisms underlying genetic diversity. Moreover, K-FluDB demonstrates exceptional versatility, validated not only with controlled datasets but also with real-world genomic samples, achieving high accuracy and specificity in these contexts. This capability underscores its potential for integration into global genomic surveillance frameworks, facilitating more effective monitoring of circulating strains and emerging threats.

In comparison to existing resources such as GenBank, RefSeq, and INSaFLU, K-FluDB offers several distinctive advantages for genomic surveillance of Influenza A viruses. Traditional databases often require full genome assembly or BLAST-based validation to infer subtypes, which increases computational burden and may introduce errors in the presence of reassortment or miss annotations. RefSeq, while curated, is limited by its small size and non-redundant design, leading to poor subtype coverage, reduced sensitivity, and missing Hx Nx subtypes. INSaFLU enables subtype detection but relies on contig reconstruction and does not quantify subtype consistency across segments. By contrast, K-FluDB supports direct classification of raw sequencing reads using subtype-specific pangenomes for each segment, avoiding the need for assembly and enabling accurate detection of unusual or reassorted constellations. The database design eliminates redundancy while retaining informative diversity, improving subtype resolution and reducing ambiguous mappings. Moreover, its segment-level structure allows reassortment detection across all eight segments, not only HA and NA, providing a broader and more precise view of viral evolution and transmission. These features collectively position K-FluDB as a scalable and efficient tool for high-throughput influenza surveillance and outbreak investigation.

## Supplementary Material

vbaf254_Supplementary_Data

## Data Availability

Three versions of K-FluDB, optimized for read lengths of 75, 150, and 300 nucleotides, are freely available at https://zenodo.org/records/17203072, and the source code is available at https://github.com/usjunco/pangen.
