# Supplementary material for: K-FluDB: a novel K-mer-based database for enhanced genomic surveillance of Influenza A viruses
Source: Bioinform Adv. 2025 Oct 10;6(1):vbaf254. doi: 10.1093/bioadv/vbaf254 (PMC13275130; doi:10.1093/bioadv/vbaf254)
Supplement: vbaf254_Supplementary_Data [file vbaf254_supplementary_data.docx]

**K-FluDB: A Novel K-Mer Based Database for Enhanced Genomic Surveillance of Influenza A Viruses**

Supplementary Tables S1, S2, S3, S4, S5 and S6

**Table S1**. Accuracy and precision metrics for Hemagglutinin subtypes (segment 4 of influenza A) of the subtype-specific K-FluDB.

| **Subtype** | **Precision** | **True Positive Rate** |
| --- | --- | --- |
| H1 | 99.95 | 99.98 |
| H2 | 100 | 99.4 |
| H3 | 99.95 | 99.95 |
| H4 | 99.92 | 100 |
| H5 | 100 | 99.94 |
| H6 | 100 | 100 |
| H7 | 99.99 | 99.96 |
| H8 | 100 | 100 |
| H9 | 100 | 100 |
| H10 | 99.67 | 98.51 |
| H11 | 97.84 | 99.7 |
| H12 | 100 | 99.56 |
| H13 | 99.55 | 99.75 |
| H14 | 100 | 100 |
| H15 | 100 | 100 |
| H16 | 99.57 | 99.14 |
| H17 | 100 | 100 |
| H18 | 100 | 100 |
| **avg** | **99.8** | **99.77** |

**Table S2**. Accuracy and precision metrics for Neuraminidase subtypes (segment 6 of influenza A) of the subtype-specific K-FluDB.

| **Subtype** | **Precision** | **True Positive Rate** |
| --- | --- | --- |
| N1 | 99.94 | 99.98 |
| N2 | 99.82 | 99.93 |
| N3 | 99.71 | 96.78 |
| N4 | 99.18 | 98.93 |
| N5 | 99.29 | 98.25 |
| N6 | 99.67 | 99.87 |
| N7 | 99.82 | 100 |
| N8 | 99.64 | 99.86 |
| N9 | 99.78 | 99.78 |
| N10 | 100 | 100 |
| N11 | 100 | 100 |
| **avg** | **99.71** | **99.4** |

###

**Table S3.** Confusion matrix with true and predicted subtypes for segment 4.

|  | **predicted subtype** | | | | | | | | | | | | | | | | | |
| --- | --- | --- | --- | --- | --- | --- | --- | --- | --- | --- | --- | --- | --- | --- | --- | --- | --- | --- |
| **true subtype** | **H1** | **H2** | **H3** | **H4** | **H5** | **H6** | **H7** | **H8** | **H9** | **H10** | **H11** | **H12** | **H13** | **H14** | **H15** | **H16** | **H17** | **H18** |
| **H1** | 562825 | 0 | 79 | 0 | 0 | 0 | 0 | 0 | 0 | 0 | 32 | 0 | 0 | 0 | 0 | 0 | 0 | 0 |
| **H2** | 0 | 10606 | 64 | 0 | 0 | 0 | 0 | 0 | 0 | 0 | 0 | 0 | 0 | 0 | 0 | 0 | 0 | 0 |
| **H3** | 241 | 0 | 537211 | 12 | 5 | 0 | 5 | 0 | 0 | 0 | 0 | 0 | 0 | 0 | 0 | 0 | 0 | 0 |
| **H4** | 0 | 0 | 0 | 32989 | 0 | 0 | 0 | 0 | 0 | 0 | 0 | 0 | 0 | 0 | 0 | 0 | 0 | 0 |
| **H5** | 8 | 0 | 27 | 0 | 101325 | 0 | 0 | 0 | 0 | 16 | 0 | 0 | 0 | 0 | 0 | 14 | 0 | 0 |
| **H6** | 0 | 0 | 0 | 0 | 0 | 29524 | 0 | 0 | 0 | 0 | 0 | 0 | 0 | 0 | 0 | 0 | 0 | 0 |
| **H7** | 0 | 0 | 6 | 0 | 0 | 0 | 50631 | 0 | 0 | 16 | 0 | 0 | 0 | 0 | 0 | 0 | 0 | 0 |
| **H8** | 0 | 0 | 0 | 0 | 0 | 0 | 0 | 2864 | 0 | 0 | 0 | 0 | 0 | 0 | 0 | 0 | 0 | 0 |
| **H9** | 0 | 0 | 0 | 0 | 0 | 0 | 0 | 0 | 65043 | 0 | 0 | 0 | 0 | 0 | 0 | 0 | 0 | 0 |
| **H10** | 32 | 0 | 62 | 16 | 0 | 0 | 0 | 0 | 0 | 19486 | 184 | 0 | 0 | 0 | 0 | 0 | 0 | 0 |
| **H11** | 0 | 0 | 0 | 0 | 0 | 0 | 0 | 0 | 0 | 32 | 10520 | 0 | 0 | 0 | 0 | 0 | 0 | 0 |
| **H12** | 0 | 0 | 0 | 0 | 0 | 0 | 0 | 0 | 0 | 0 | 16 | 3657 | 0 | 0 | 0 | 0 | 0 | 0 |
| **H13** | 0 | 0 | 16 | 0 | 0 | 0 | 0 | 0 | 0 | 0 | 0 | 0 | 8375 | 0 | 0 | 5 | 0 | 0 |
| **H14** | 0 | 0 | 0 | 0 | 0 | 0 | 0 | 0 | 0 | 0 | 0 | 0 | 0 | 447 | 0 | 0 | 0 | 0 |
| **H15** | 0 | 0 | 0 | 0 | 0 | 0 | 0 | 0 | 0 | 0 | 0 | 0 | 0 | 0 | 330 | 0 | 0 | 0 |
| **H16** | 0 | 0 | 0 | 0 | 0 | 0 | 0 | 0 | 0 | 0 | 0 | 0 | 38 | 0 | 0 | 4379 | 0 | 0 |
| **H17** | 0 | 0 | 0 | 0 | 0 | 0 | 0 | 0 | 0 | 0 | 0 | 0 | 0 | 0 | 0 | 0 | 51 | 0 |
| **H18** | 0 | 0 | 0 | 0 | 0 | 0 | 0 | 0 | 0 | 0 | 0 | 0 | 0 | 0 | 0 | 0 | 0 | 34 |

**Table S4.** Confusion matrix with true and predicted subtypes for segment 6.

|  | **predicted subtype** | | | | | | | | | | |
| --- | --- | --- | --- | --- | --- | --- | --- | --- | --- | --- | --- |
| **true subtype** | **N1** | **N2** | **N3** | **N4** | **N5** | **N6** | **N7** | **N8** | **N9** | **N10** | **N11** |
| **N1** | 377176 | 58 | 0 | 13 | 0 | 0 | 0 | 13 | 0 | 0 | 0 |
| **N2** | 195 | 384636 | 25 | 0 | 0 | 40 | 0 | 26 | 0 | 0 | 0 |
| **N3** | 0 | 593 | 20414 | 0 | 0 | 60 | 13 | 0 | 13 | 0 | 0 |
| **N4** | 0 | 12 | 0 | 4715 | 39 | 0 | 0 | 0 | 0 | 0 | 0 |
| **N5** | 26 | 13 | 0 | 0 | 7301 | 13 | 0 | 52 | 26 | 0 | 0 |
| **N6** | 0 | 0 | 8 | 0 | 0 | 37638 | 0 | 27 | 13 | 0 | 0 |
| **N7** | 0 | 0 | 0 | 0 | 0 | 0 | 14180 | 0 | 0 | 0 | 0 |
| **N8** | 0 | 0 | 13 | 26 | 0 | 13 | 0 | 36362 | 0 | 0 | 0 |
| **N9** | 0 | 0 | 13 | 0 | 13 | 0 | 13 | 13 | 23992 | 0 | 0 |
| **N10** | 0 | 0 | 0 | 0 | 0 | 0 | 0 | 0 | 0 | 39 | 0 |
| **N11** | 0 | 0 | 0 | 0 | 0 | 0 | 0 | 0 | 0 | 0 | 26 |

**Table S5**. Compression times for each Influenza A segment for each compression k-mer length.

| **Segment** | **Number of sequences** | **k-mers in compression procedure** | **Total CPU time**  **(segs) 2 threads**  **k = 75** | **Total CPU time**  **(segs) 2 threads**  **k = 150** | **Total CPU time**  **(segs) 2 threads**  **k = 300** |
| --- | --- | --- | --- | --- | --- |
| 1 (PB2) | 9,811 | 402,330 | 205.802s | 2219.223s | 5796.157s |
| 2 (PB1) | 9,946 | 407,784 | 341.117s | 2872.803s | 7374.834s |
| 3 (PA) | 9,868 | 387,015 | 419.941s | 2483.495s | 6555.666s |
| 4 (HA) | 11,287 | 322,622 | 593.748s | 1188.964s | 2035.830s |
| 5 (NP) | 9,872 | 259,718 | 611.955s | 1559.722s | 3911.835s |
| 6 (NA) | 10,478 | 253,067 | 843.207s | 976.374s | 1954.319s |
| 7 (M1, M2) | 10,056 | 163,092 | 864.322s | 811.832s | 1575.998s |
| 8 (NS1, NEP) | 9,944 | 134,384 | 946.758s | 667.717s | 1413.718s |
